# Supplementary material for: Dominant control of temperature on (sub-)tropical soil carbon turnover
Source: Nat Commun. 2025 May 15;16:4530. doi: 10.1038/s41467-025-59013-9 (PMC12081612; doi:10.1038/s41467-025-59013-9)
Supplement: Supplementary file 1 — Supplementary Information [file 41467_2025_59013_MOESM1_ESM.pdf]

Supplementary material for:

Dominant control of temperature on (sub-)tropical soil carbon turnover

Vera D. Meyer[1*], Peter Köhler[2], Nadine T. Smit[1,3], Julius S. Lipp[1], Bingbing Wei[2], Gesine Mollenhauer[1,2,4] and Enno Schefuß[1*]

[1]: MARUM – Center for Marine Environmental Sciences, University of Bremen, Germany

[2]: Alfred-Wegener-Institut Helmholtz Zentrum für Polar- und Meeresforschung, Bremerhaven, Germany

[3]: now at: Bruker Daltonics GmbH & Co. KG., Bremen, Germany

[4]: Department of Geosciences, University of Bremen, Bremen, Germany

*corresponding authors: vmeyer@marum-alumni.de; eschefuss@marum.de

Contents

Supplementary Figures

Supplementary Tables

Supplementary References

Supplementary Figures

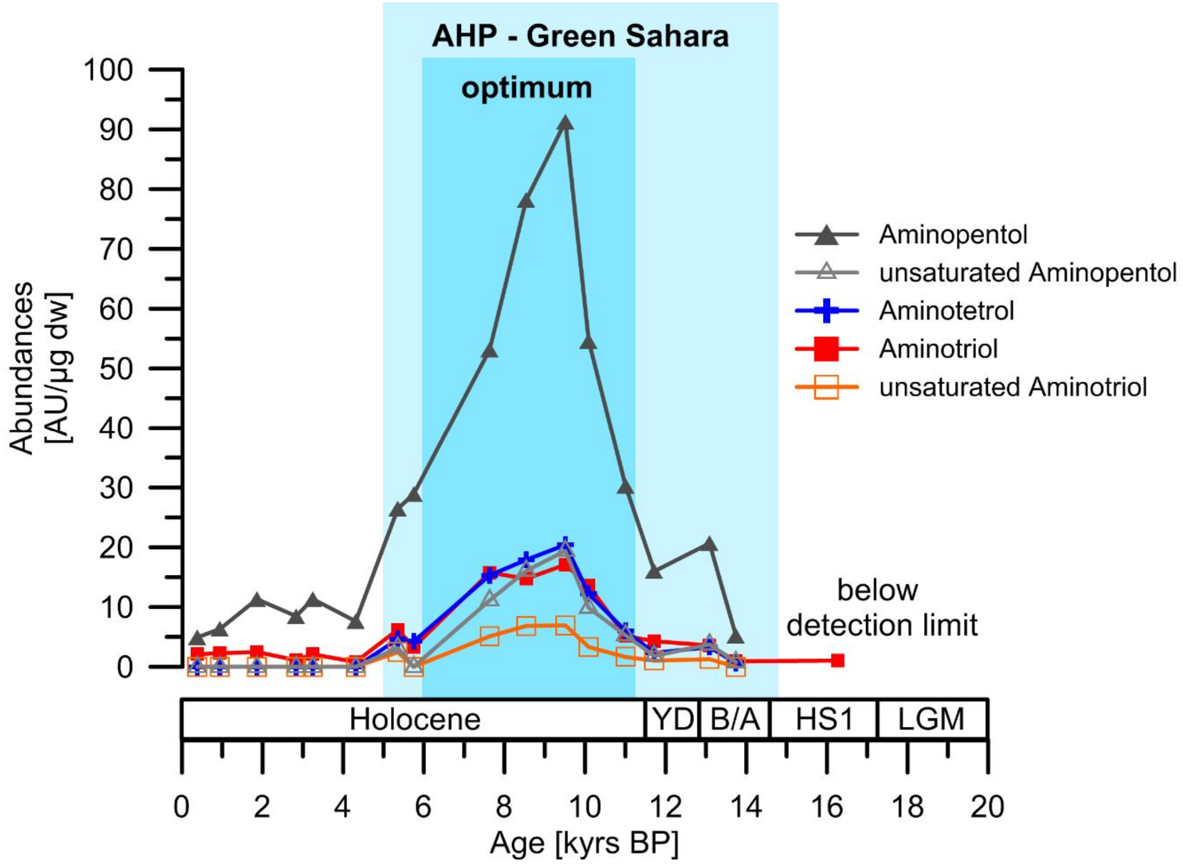

Figure 1. **Abundances of amino-bacteriohopanepolyols in core GeoB7702-3.** The contents are normalized to the dry weight of extracted sediment (µg dw). AU: Arbitrary units. The blue bars mark the timing of the African Humid Period (AHP), "Green Sahara" and their optimum[1,2.] LGM: Last Glacial Maximum; HS1: Heinrich Stadial 1; B/A: Bølling/Allerød interstadial; YD: Younger Dryas stadial.

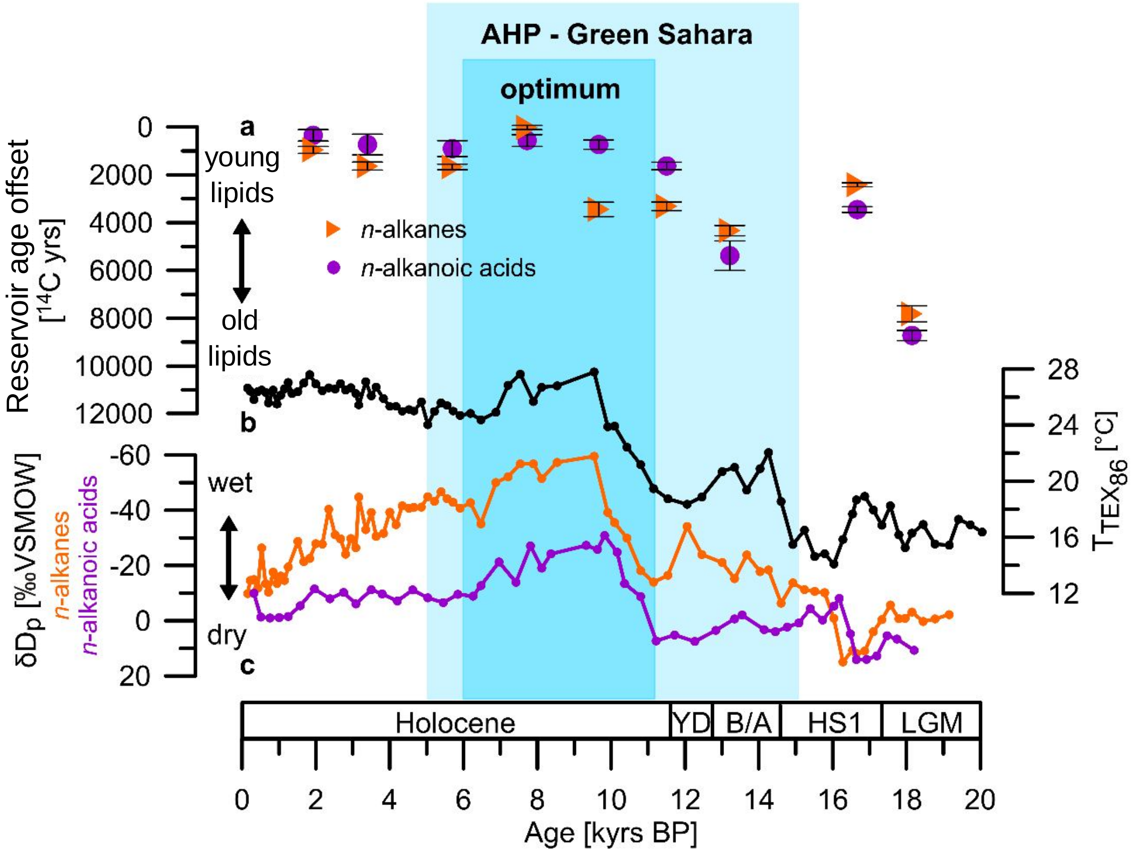

Figure 2. **Climate reconstructions for the Nile River catchment and reservoir age offsets between leaf-wax lipids and the atmosphere at the time of deposition at site GeoB7702-3. a** Reservoir age offsets (R) of *n*-alkanoic acids (dots) and *n*-alkanes (triangles) calculated after ref.[3]. For *n*-alkanoic acids R is based on the compound-specific radiocarbon analysis (CSRA) of the *n*-$C_{26:0}$ and *n*-$C_{28:0}$ homologues. R of *n*-alkanes is calculated from CSRA of the *n*-C_{29}, *n*-C_{31}, *n*-C_{33} alkane homologues. Error bars represent the standard deviation. R is a measure for the age of *n*-alkanoic acids and *n*-alkanes at the time of deposition in marine sediments at the core site. **b** Sea surface temperature reconstruction for the eastern Mediterranean based on the TEX_{86}-proxy (T_{TEX86}) in core GeoB7702-3 from ref.[4]. **c** Hydrogen isotope compositions of precipitation (δD_p) calculated from δD of leaf-wax lipids, orange: *n*-C_{31} alkane from ref.[5] and violet: mean of *n*-$C_{26:0}$ and *n*-$C_{28:0}$ *n*-alkanoic acids from ref.[6] in core GeoB7702-3. δD_p is calculated against the Vienna Standard Mean Ocean Water (VSMOW). The blue bars mark the timing of the African Humid Period (AHP), "Green Sahara" and their optimum[1,2]. LGM: Last Glacial Maximum; HS1: Heinrich Stadial 1; B/A: Bølling/Allerød interstadial; YD: Younger Dryas stadial.

Supplementary Tables

Table 1. **Results of compound-specific radiocarbon analysis (CSRA) in core GeoB7702-3.** CSRA was performed on the n-$C_{26:0}$ and n-$C_{28:0}$ alkanoic acid and the n-C_{29}, n-C_{31} and n-C_{33} alkane homologues at the Alfred Wegener Institut Helmholtz Zentrum für Polar und Meeresforschung, Bremerhaven, Germany (AWI) by Accelerator Mass Spectrometry (AMS) using the Ionplus MICADAS-system equipped with a gas-ion source. The sample identification numbers are provided (AWI sample ID). Radiocarbon contents are expressed in the fraction modern carbon ($F^{14}C$) and $\Delta^{14}C$ notations as defined in ref.[7]. AMS results were corrected for procedure blanks after ref.[8]. R is the reservoir age offset between the biomarkers and the atmosphere at the time of deposition in marine sediments at site GeoB7702-3 calculated after ref.[3]. The standard deviation (±) is reported along with the results.

Sample depth [cm]	AWI sample ID	Deposition age range [kyrs BP][a]	Deposition age mid-point [kyrs BP][a]	Compounds	$F^{14}C$[b]	$\Delta^{14}C$[b] [‰]	R [^{14}C yrs]
81.5-84.5	5252.1.1	1.62 - 2.29	1.93	n-$C_{26:0}$ + n-$C_{28:0}$	0.7408 ± 0.0281	-265 ± 28	348 ± 240
81.5-84.5	5252.2.1	1.62 - 2.29	1.93	n-C_{29} + n-C_{31} + n-C_{33}	0.6866 ± 0.0158	-319 ± 16	959 ± 146
130-133	5061.2.1	3.11 - 3.69	3.40	n-$C_{26:0}$	0.6180 ± 0.0130	-387 ± 13	604 ± 112
130-133	5061.3.1	3.11 - 3.69	3.40	n-$C_{28:0}$	0.5975 ± 0.0142	-408 ± 14	875 ± 126
130-133	-	3.11 - 3.69	3.40	n-$C_{26:0}$ + n-$C_{28:0}$[c]	0.6082 ± 0.0498[c]	-397 ± 50	733 ± 432
130-133	5061.4.1	3.11 - 3.69	3.40	n-C_{29} + n-C_{31} + n-C_{33}	0.5437 ± 0.0172	-461 ± 17	1633 ± 167
198-201	5251.2.1	5.35 - 6.01	5.70	n-$C_{26:0}$	0.4795 ± 0.0131	-525 ± 13	871 ± 111
198-201	5251.1.1	5.35 - 6.01	5.70	n-$C_{28:0}$	0.4761 ± 0.0152	-528 ± 15	929 ± 129
198-201	-	5.35 - 6.01	5.70	n-$C_{26:0}$ + n-$C_{28:0}$[c]	0.4777 ± 0.0395[c]	-526 ± 40	902 ± 331
198-201	5251.3.1	5.35 - 6.01	5.70	n-C_{29} + n-C_{31} + n-C_{33}	0.4342 ± 0.0125	-569 ± 13	1668 ± 116
231-234	11116.2.1	7.24 - 8.14	7.72	n-$C_{26:0}$	0.4086 ± 0.0085	-595 ± 9	202 ± 69
231-234	11116.3.1	7.24 - 8.14	7.72	n-$C_{28:0}$	0.3705 ± 0.0092	-633 ± 9	988 ± 81
231-234	-	7.24 - 8.14	7.72	n-$C_{26:0}$ + n-$C_{28:0}$[c]	0.3906 ± 0.0307[c]	-613 ± 31	563 ± 247
231-234	11116.1.1	7.24 - 8.14	7.72	n-C_{29} + n-C_{31} + n-C_{33}	0.4179 ± 0.0112	-586 ± 11	21 ± 87
251-254	5060.2.1	9.02 - 10.11	9.66	n-$C_{26:0}$	0.3062 ± 0.0093	-696 ± 9	734 ± 79
251-254	5060.3.1	9.02 - 10.11	9.66	n-$C_{28:0}$	0.3060 ± 0.0085	-697 ± 8	738 ± 79
251-254	-	9.02 - 10.11	9.66	n-$C_{26:0}$ + n-$C_{28:0}$[c]	0.3061 ± 0.0240[c]	-696 ± 24	736 ± 196
251-254	5060.4.1	9.02 - 10.11	9.66	n-C_{29} + n-C_{31} + n-C_{33}	0.2184 ± 0.0263	-783 ± 26	3447 ± 298
278-281	5059.2.1	11.05 - 12.05	11.50	n-$C_{26:0}$	0.2183 ± 0.0082	-784 ± 8	2126 ± 77
278-281	5059.4.1	11.05 - 12.05	11.50	n-$C_{28:0}$	0.2475 ± 0.0088	-755 ± 9	1117 ± 74
278-281	-	11.05 - 12.05	11.50	n-$C_{26:0}$ + n-$C_{28:0}$[c]	0.2321 ± 0.0183[c]	-770 ± 18	1613 ± 158
278-281	5059.5.1	11.05 - 12.05	11.50	n-C_{29} + n-C_{31} + n-C_{33}	0.1883 ± 0.0167	-813 ± 17	3313 ± 178
297-300	5250.1.1	12.69 - 13.73	13.21	n-$C_{26:0}$ + n-$C_{28:0}$	0.1236 ± 0.0474	-877 ± 47	5384 ± 618
297-300	5250.2.1	12.69 - 13.73	13.21	n-C_{29} + n-C_{31} + n-C_{33}	0.1408 ± 0.0185	-860 ± 19	4334 ± 213
359-362	5249.2.1	16.26 - 17.07	16.67	n-$C_{26:0}$	0.1118 ± 0.0160	-889 ± 16	3763 ± 157
359-362	5249.1.1	16.26 - 17.07	16.67	n-$C_{28:0}$	0.1206 ± 0.0158	-880 ± 16	3154 ± 144
359-362	-	16.26 - 17.07	16.67	n-$C_{26:0}$ + n-$C_{28:0}$[c]	0.1162 ± 0.0123[c]	-885 ± 12	3453 ± 219
359-362	5249.3.1	16.26 - 17.07	16.67	n-C_{29} + n-C_{31} + n-C_{33}	0.1322 ± 0.0089	-869 ± 9	2415 ± 81
393-396	5248.2.1	17.69 - 18.73	18.15	n-$C_{26:0}$	0.0748 ± 0.0269	-926 ± 27	6005 ± 322
393-396	5248.1.1	17.69 - 18.73	18.15	n-$C_{28:0}$	0.0372 ± 0.0403	-963 ± 40	11611 ± 961
393-396	-	17.69 - 18.73	18.15	n-$C_{26:0}$ + n-$C_{28:0}$[c]	0.0534 ± 0.0125[c]	-947 ± 12	8723 ± 212
393-396	5248.3.1	17.69 - 18.73	18.15	n-C_{29} + n-C_{31} + n-C_{33}	0.0597 ± 0.0228	-941 ± 23	7816 ± 341

a: Obtained from the core chronology which is based on radiocarbon dating of planktic foraminifera[6].

b: Corrected for procedure blanks after ref.[8]. n-$C_{26:0}$ and n-$C_{28:0}$ alkanoic acids were additionally corrected for the carbon introduced during methylation.

c: Calculated abundance-weighted means of the n-$C_{26:0}$ and n-$C_{28:0}$ homologue.

Supplementary References

1. DeMenocal, P. *et al.* Abrupt onset and termination of the African Humid Period: Rapid climate responses to gradual insolation forcing. *Quat. Sci. Rev.* **19**, 347–361 (2000).
2. Kuper, R. & Kröpelin, S. Climate-controlled Holocene occupation in the Sahara: Motor of Africa's evolution. *Science* **313**, 803–807 (2006).
3. Soulet, G., Skinner, L. C., Beaupré, S. R. & Galy, V. A note on reporting of reservoir ^{14}C disequilibria and age offsets. *Radiocarbon* **58**, 205–211 (2016).
4. Castañeda, I. S. *et al.* Millennial-scale sea surface temperature changes in the eastern Mediterranean (Nile River Delta region) over the last 27,000 years. *Paleoceanography* **25**, 1–13 (2010).
5. Castañeda, I. S. *et al.* Hydroclimate variability in the Nile River Basin during the past 28,000 years. *Earth Planet. Sci. Lett.* **438**, 47–56 (2016).
6. Meyer, V. D. *et al.* Evolution of winter precipitation in the Nile river watershed since the last glacial. *Clim. Past* **20**, 523–546 (2024).
7. Stuiver, M. & Polach, H. Discussion: reporting of ^{14}C data. *Radiocarbon* **19**, 355–363 (1977).
8. Sun, S. *et al.* ^{14}C Blank Assessment in Small-Scale Compound-Specific Radiocarbon Analysis of Lipid Biomarkers and Lignin Phenols. *Radiocarbon* **62**, 207–218 (2020).
